# Effects of Guar Gum and Sodium Benzoate on the Properties and Hydrophilicity of Silk Fibroin Hydrogels

**DOI:** 10.3390/polym17030425

**Published:** 2025-02-06

**Authors:** Ansaya Thonpho, Yodthong Baimark, Suchai Tanisood, Prasong Srihanam

**Affiliations:** Biodegradable Polymers Research Unit, Centre of Excellence for Innovation in Chemistry, Department of Chemistry, Faculty of Science, Mahasarakham University, Mahasarakham 44150, Thailand; ansaya.t@msu.ac.th (A.T.); yodthong.b@msu.ac.th (Y.B.); suchai.t@msu.ac.th (S.T.)

**Keywords:** guar gum, hydrogel, property, silk fibroin, sodium benzoate

## Abstract

Silk fibroin (SF)–based hydrogels were prepared by the simple evaporation method. The outcomes of SF–based hydrogels were assessed for consideration in terms of practical and convenient use. Guar gum (GG) and sodium benzoate (SB) are blending reagents to the SF solution and are poured into the petri dish to make the hydrogels. After leaving the mixture solution for three days to solidify, all SF–based hydrogels were peeled off and characterized. The SF–blend guar gum (SF–GG) and SF–GG–blend sodium benzoate (SF–GG–SB) could be constructed, but in different textures and levels of transparency. The SB affected the solid texture and resulted in a higher water contact angle (WCA) value of the prepared SF hydrogel than of the SF–GG. The results from Fourier transform infrared spectroscopy (FTIR) indicated all the main functional groups of substances that were contained in the blending hydrogels. Moreover, some interactions between the functional groups were also detected. A thermogravimetric analyzer (TGA) was used to determine the hydrogel decomposition as a function of temperature. The DTG thermograms, which exhibit the maximum decomposition temperature, revealed that the interaction forces between blending substances and SF, as well as their structure, are the reason for the thermal stability of the SF–based hydrogels. SF–GG–SB hydrogels have higher tensile strength than the SF–GG hydrogels. In conclusion, the appearance, texture, hydrophilicity, thermal stability, and tensile strength of the SF–based hydrogels were affected by the types and concentrations of the blending substances. This suggests that the SF–based hydrogel properties could be designed and adjusted to attain desirable textures for fitting target applications.

## 1. Introduction

Today, the search for sustainable material alternatives to synthetic polymers derived from fossil resources is becoming increasingly focused due to their environmental impact [1,2,3]. The new generation is interested in and concerned about environmentally friendly products worldwide. Natural biopolymers have been studied and applied since they are cost-effective functional materials, and environmentally safe [4,5]. In addition, natural biopolymers are sustainable, biocompatible, and biodegradable materials and their unique hierarchical structures further enhance their appeal for a wide range of uses [6,7,8]. Polysaccharides, such as cellulose [9,10,11], starch [12], alginate [13], chitosan [14], and guar gum [15] are also popularly used for several applications. Other biopolymer, protein-based materials are regularly applied in different forms and in various fields [16,17,18].

Natural silk fibers derived from silkworm cocoons have been used in textile production because of their luster and mechanical strength [19]. Silk fibroin (SF), and a glue–like protein sericin (SE) which covers SF, are the main silk components [20,21]. In the degumming process, SE is removed from cocoons or silk fabrics and usually discarded as waste, especially in the industrial production of silk fabrics [22]. This relates to environmental problems [19,23]. Previous reports showed that different silk products have been developed for application because of their excellent biological and mechanical properties [19,23,24].

Due to their biological characteristics, natural gums have drawn a lot of attention for use in raising the viscosity of solutions [15]. Plants, microbes, marine algae, and animals are among the various sources of natural gums [25,26]. During their development, plant gums were created in reaction to mechanical harm or as a defense against microbial invasions [27]. Guar gum (GG) and other plant seed gums are extensively utilized in many different industries worldwide. Reports on GG–based materials in many fields have been developed. According to Srivastava and Kapoor (2005) [28], GG is obtained from the seeds of the drought-resistant plant *Cyamopsis tetragonoloba*. GG comes in various types based on its impurity level, dissolution rate, and viscosity. Mannopyranosyl and galactopyranosyl units make up the galactomannan’s polysaccharide, which is the backbone of GG’s chemical structure. Glycosidic bonds β–d–(1 → 4) connect the linear chain mannose units, while α–d-(1 → 6) connects the side chain galactose units. Along the mannan main chain, the galactose side groups are distributed randomly [29]. The maximum viscosity of GG is attained at pH 6–9 and is extremely affected by temperature [30]. Moreover, the hydration rate of GG is affected by its concentration and particle size [31].

In recent years, SF–based devices have been taken into consideration for potential applications, especially in medical and drug delivery systems [32,33,34]. This is because of SF’s biodegradable and biocompatible characteristics [35,36]. SF microcarrier structures have been attractively developed [24]. Because of their many benefits for drug delivery systems, SF hydrogels have drawn attention from all over the world [36,37]. SF hydrogel has been used in a variety of ways in recent years [38,39,40]. In addition to its structure, hydrogel properties like flexibility, plasticity, and permeability are frequently highlighted.

According to published research, combining biopolymers offers numerous benefits for getting around their drawbacks and producing a wide range of uses. Because of their many properties and adaptability, SF and GG are promising materials. Because of its numerous functional groups, GG can readily combine with other substances to form new biomaterials for use. Blending GG with SF can serve various functional purposes depending on the intended application, especially in fields like biomaterials, food, or pharmaceuticals. It is a natural thickening agent. Therefore, GG can improve viscosity and provide a gel–like consistency, which can be useful in various formulations like films, hydrogels, or coatings. It is biocompatible and can enhance the mechanical properties of SF–based materials, making them more suitable for use in biomedical applications such as wound dressings or drug delivery systems. In addition, it can also help stabilize the fibroin structure and reduce brittleness, enhancing the material’s performance.

Sodium benzoate (SB), a salt of benzoic acid, has been mixed with biomaterials due to its antifungal and antibacterial properties and water solubility. It does not accumulate in the body [41]. It has also been mixed with biopolymers like chitosan and SF to make hydrogel for wound healing and coagulation management. When combined with SF, it can help keep the material free from microbial contamination, which is important in biomedical or food–related applications. It can also act as an antioxidant, potentially increasing the durability and stability of SF–based products [42]. In general, plasticizers like glycerol are commonly used in polymer-based materials. It can make the SF more flexible and less brittle, improving its mechanical properties for application [43]. Moreover, it also helps in retaining moisture, which can be crucial in applications like wound dressings or skin care products, where maintaining hydration is important.

The development of SF–based hydrogels is an attractive issue of great significance to broadening their applications. Their ability to retain moisture, be biocompatible, and carry bioactive substances makes them versatile materials for a variety of innovative and sustainable uses across biomedicine (wound dressings), cosmetics, food, agriculture, bioadhesives, and even wearable electronics [44,45,46]. The hydrogels can retain water and adjust to make various shapes, which are suitable for creating responsive materials and sensors [47]. Moreover, SF–based hydrogels have been used in tissue engineering [15], the postoperative rehabilitation of cancer patients [48], and enhanced neuroregeneration [49]. SF hydrogels, especially when blended with materials like GG, SB, and glycerol, have a range of potential applications, particularly in fields where biocompatibility, flexibility, and moisture retention are crucial.

In this work, SF aqueous solution was prepared from cocoons after degumming. The prepared SF solution was used as the main substance to prepare SF–based hydrogels. The SF solution was blended with GG, SB, and glycerol, then stirred to homogeneity before pouring into the culture plates to make the hydrogel. Afterward, the solution mixture was left for 3 days to solidify. The texture and appearance of the SF–based hydrogels were assessed following their separation from the plates. The degradation behavior of the prepared SF–based hydrogels in different media was examined. To further elucidate the effects of each blending substance on the produced SF–based hydrogels, hydrophilicity, strength, thermal stability, and conformational change were investigated.

## 2. Materials and Methods

### 2.1. Materials

The cocoons of *Bombyx mori* were obtained from the Center of Excellence for Silk Innovation at Mahasarakham University, located in the Khamriang sub-district of Kantharawichai, Maha Sarakham, Thailand. We obtained ethanol (C_2_H_5_OH), sodium carbonate (Na_2_CO_3_), calcium chloride (CaCl_2_) and sodium chloride (NaCl) from the Merck KGaA company (Darmstadt, Germany) and Ajax Finechem Pty Ltd. (Auckland, New Zealand). Before use, none of the reagent-grade chemicals used in this study required additional purification. Guar gum was purchased from commercial trade in Thailand. Sodium benzoate (C_7_H_5_O_2_Na) and glycerol (C_3_H_8_O_3_) were supplied from the Merck KGaA company (Darmstadt, Germany)

### 2.2. Preparation of SF Solution

The Thai strain of *B. mori* cocoons were cleaned and cut into small pieces. They were then twice boiled for 30 min each at 100 °C in a 0.5% (*w*/*v*) Na_2_CO_3_ solution to remove the glue–like protein, sericin (SE). To obtain SF, the degummed cocoon samples were rinsed with distilled water until the pH level became neutral. After that, a tertiary solvent system consisting of CaCl_2_:C_2_H_5_OH:H_2_O (1:2:8 by mol) was used to dissolve the degummed cocoon samples for 60 min at 75 °C while stirring continuously. The hydrolysate SF thus obtained was dialyzed against distilled water for three days to remove any salt using a dialysis membrane (MW cut off 10 kDa, Thermo Fisher Scientific Inc., Waltham, MA, USA). After the SF solution’s concentration was determined, distilled water was added to dilute it to 2% (*w*/*v*).

### 2.3. Preparation of SF–Based Hydrogels

Twenty mL of SF solution was blended with different substances to find a favorite composition of the component, including various concentrations of guar gum (GG) (0.1–0.4 g) and sodium benzoate (SB) (0.1–0.4 g). After mixing, the mixture was stirred until a homogeneous mixture was obtained and then poured into the polystyrene culture plates with a 9 cm diameter. All samples were air-dried for several days at room temperature to obtain hydrogel–based SF for further characterization.

### 2.4. Characteristics of SF–Based Hydrogels

#### 2.4.1. Transparency Observation

As mentioned previously [50], a UV–Vis spectrophotometer (Lambda 25, Perkin Elmer, MA, USA) was used to measure the transparency of the SF–based hydrogels that were constructed. In short, rectangular pieces of the hydrogels were cut out and put straight into the spectrophotometer cell. The average transparency value was then determined by measuring the percentage transmittance of light at 660 nm through each hydrogel three times.

#### 2.4.2. Analysis of Functional Groups

Utilizing an attenuated reflection–Fourier transform infrared (ATR–FTIR) spectrometer (Invenio-S, Bruker, Karlsruhe, Germany), the functional groups of the SF–based hydrogels were examined. The FTIR spectrum results were acquired with 32 scans and a wavenumber range of 4000–400 cm^−1^ at a spectral resolution of 4 cm^−1^. Air served as the reference for this procedure. 

#### 2.4.3. Thermal Stability

The thermal stability of the constructed SF–based hydrogels was investigated using a thermogravimetric analyzer (TGA) (SDTQ600, TA-Instrument Co., Ltd., New Castle, DE, USA). The samples were placed within an aluminum pan before heating in the range of 50 to 800 °C by fixing a rate of 20 °C per minute. The condition of the process was performed in a nitrogen environment. The decreases in weight were recorded at several points in time.

#### 2.4.4. Mechanical Properties

Using the tensile testing machine and the ASTM D638 testing procedure, the mechanical characteristics of SF–based hydrogels were assessed. The samples were fixed to the machine using tensile grips after being cut into rectangular pieces measuring 200 mm by 50 mm. At room temperature, a testing speed of 2 mm/min was employed. A computer was used to monitor and control the process. The stress–strain curve was used to determine the tensile strength (MPa) and elongation at break. For each, three specimens were inspected for mechanical alterations.

#### 2.4.5. Degradation of SF–based Hydrogels

The degradation behavior of the SF–based hydrogels in different media (phosphate buffer at pH 7.4, with 1.0 M HCl, 1.0 M NaOH, 95% C_2_H_5_OH, and 1.0 M NaCl solution) were performed following the method of the previous report [18] with slight modifications. Firstly, the dry and clean glass vials were numbered, and the total mass of the glass vials was weighed. The SF–based hydrogels were added in the vials and weighed. The degradation experiment was conducted at 37 °C in an incubator after the fresh degradation solution was added at a 1:1 (*v*/*v*) ratio to the glass vial containing the hydrogel. The new degradation solution was changed daily throughout the course of the experiments. The samples were removed, and the degradation solution was disposed of once the predetermined time had passed. Weighing was conducted to determine the combined mass of the glass vial and the leftover hydrogel. Equation (1) was used to determine the hydrogel’s residual mass-retention rate [24].

Remaining mass retention rate (%) = (Mi − M)/(M0 − M) × 100(1)

where M0 represents the initial mass (g) of the glass vial and hydrogel, M is the glass vial’s mass (g), and Mi is the mass (g) of the glass vial and any hydrogel that remains after i days.

#### 2.4.6. Hydrophilicity Test

The hydrophilicity of the SF–based hydrogel surfaces was determined utilizing a WCA analyzer (model OCA 11, DataPhysics Instruments GmbH, Filderstadt, Germany). The SF–based hydrogels were separated into 3 × 5 cm^2^ rectangles and placed on a moving, horizontal platform that had been covered in black Teflon in a WCA analyzer. A suitable droplet of water (5–10 μL) was applied to the hydrogel’s surface using a microsyringe. The contact angle of the water droplet was measured. The averages of each sample’s triplicate measurements were calculated for the results.

## 3. Results and Discussion

### 3.1. Appearance Features

#### 3.1.1. SF–GG–Hydrogels

**Figure 1:**
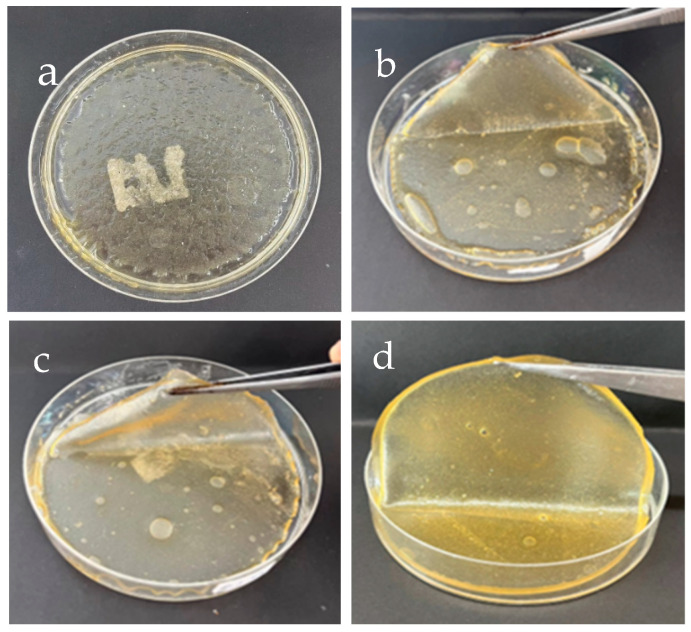
Appearance of SF hydrogels mixed with different GG contents: SF–GG (0.1) (**a**), SF–GG (0.2) (**b**), SF–GG (0.3) (**c**), and SF–GG (0.4) (**d**).

SF mixed with different concentrations of GG (0.1–0.4 g/mL) (SF–GG) was prepared to make hydrogels, as indicated in Figure 1. The obtained SF–GG hydrogels have a high moisture content and are translucent. The SF–GG (0.1) hydrogel (Figure 1a) has a thin, smooth surface and is highly translucent, but it does not form a complete texture and cannot be peeled off from the plate. The SF–GG (0.2) hydrogel (Figure 1b) clung together to form a sheet. Though it is quite soft and folds easily, the prepared hydrogel can be peeled off from the plate. When GG content was increased, the SF–GG (0.3) hydrogel, which has smoother surfaces and is more rigid, peeled off with ease (Figure 1c). All things considered, this ratio works best for producing SF–GG hydrogel that can be used and transported. Although it is thicker and less transparent, the resulting SF–GG (0.4) (Figure 1d) shares most of the same properties as the SF–GG (0.3) hydrogel.

#### 3.1.2. SF–GG (0.3) Mixed SB Hydrogels

**Figure 2:**
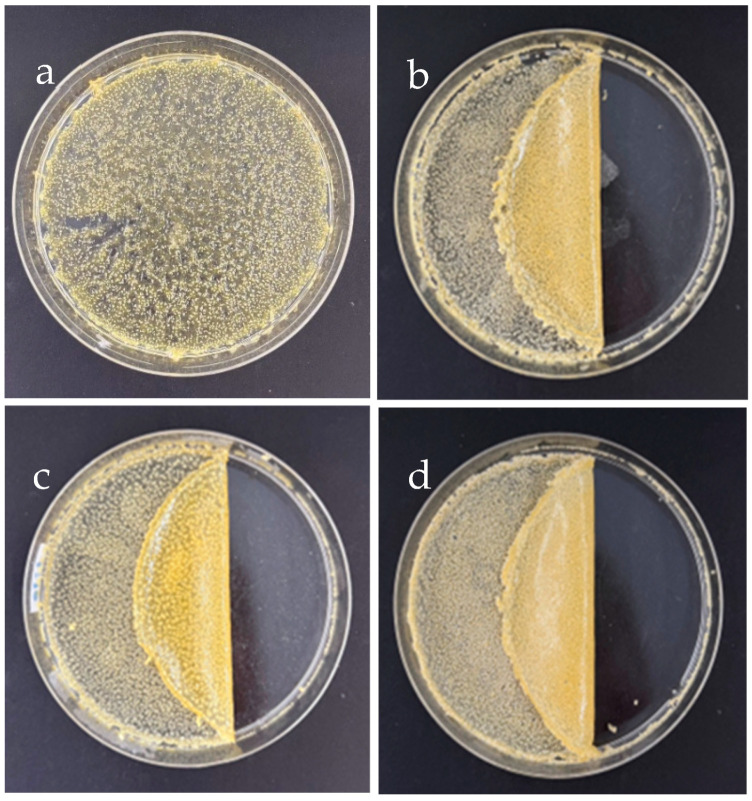
Appearance of SF–GG (0.3) hydrogels mixed with different SB contents: SF–GG (0.3)–SB (0.1) (**a**), SF–GG (0.3)–SB (0.2) (**b**), SF–GG (0.3)–SB (0.3) (**c**), and SF–GG (0.3)–SB (0.4) (**d**).

As seen in Figure 2, the SF mixed with 0.3 g/mL (SF–GG (0.3)) was chosen for blending with various SB concentrations (0.1–0.4 g/mL) because it showed the best appearance. In general, SB particles are dispersed throughout the hydrogel’s surface. The SF–GG (0.3)–SB (0.1) hydrogel (Figure 2a) remains intact and does not detach from the plate. The blended hydrogel came together as a sheet when the SB concentration was raised (SF–GG (0.3)–SB (0.2)) (Figure 2b), but it was thin and highly translucent. The resulting hydrogel is harder and stickier when the amount of SB is increased (SF–GG (0.3)–SB (0.3)) (Figure 2c). The surface of the prepared hydrogel was smooth and shiny, especially the side contacting the plate. As for the SF–GG (0.3)–SB (0.4) (Figure 2d), the obtained hydrogel was generally like the SF–GG (0.3)–SB (0.3) hydrogel but was thicker and had lower transparency. Overall, the SF–GG (0.3)–SB (0.2–0.4) resulted in suitably textured hydrogels for application. The suitable hydrogel texture should be formed with homogeneous sheets that are reasonably thick and can be moved easily. In addition, transparency and water retention are also desirable properties. The latter two properties are relevant for their use, especially wound dressings and tissue engineering.

### 3.2. Transparency and Degradation of the Hydrogel–Based SF

**Figure 3:**
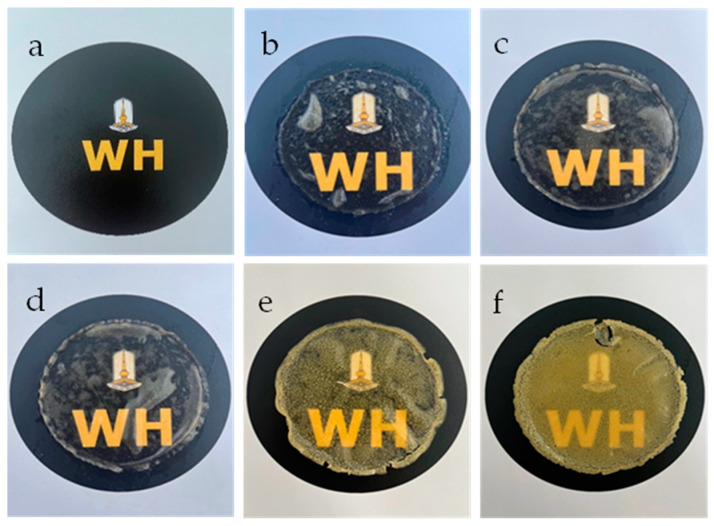
Different appearances of SF–GG (0.2) (**b**), SF–GG (0.3) (**c**), SF–GG (0.4) (**d**), SF–GG (0.3)–SB (0.3) (**e**), and SF–GG (0.3)–SB (0.4) (**f**). The letters without hydrogel (**a**) as comparison.

The transparency of hydrogels was easily checked by placing the prepared hydrogels on top of letters. After that, the clarity of the letters below was observed as seen in Figure 3. As SF–GG hydrogels, they have an overall appearance that is white, slightly glossy, and extremely transparent (Figure 3b–d). The homogeneous glycerol-plasticized texture indicated that the glycerol was evenly distributed and contributed to the blending of the various molecules. The SF–GG (0.3)–SB (0.3) (Figure 3e) and SF–GG (0.3)–SB (0.4) (Figure 3f) hydrogels are less transparent and have less moisture than SF–GG hydrogels. As the concentration of SB increased, transparency decreased. The transparency of all prepared hydrogels is lower than that of the hydrogel-free sample (Figure 3a).

All prepared SF–based hydrogels were tested for light transmittance (T660), which reflected the transparency of the hydrogels. As shown in Table 1, the highest transmittance value of 65.30% was found in the hydrogel prepared by SF–GG (0.2); this value decreased gradually with an increase in GG contents. Conversely, the lowest light transmittance value obtained was from the hydrogel which was prepared from SF–GG (0.3)–SB (0.4). From these results, it can be noted that the hydrogel transparency gradually decreased when contents of both GG and SB were increased. However, SB impacted highly on the rigid texture of the hydrogels. When a substance is mixed in larger quantities, less light can pass through because more light will be absorbed and scattered by the mixture’s molecules or particles. Combining various materials would result in new interactions that would reorganize the hydrogel texture’s three-dimensional network [51].

**Figure 4:**
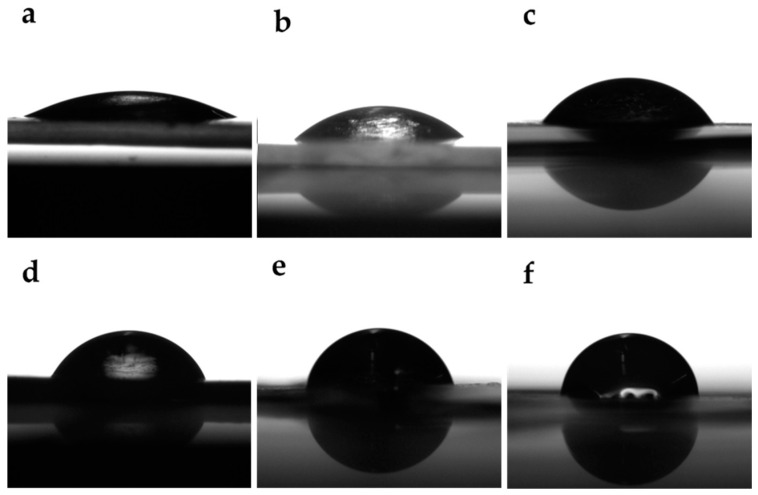
Water contact angles of hydrogels; SF–GG (0.2) (**a**), SF–GG (0.3) (**b**), SF–GG (0.4) (**c**), SF–GG (0.3)–SB (0.2) (**d**), SF–GG (0.3)–SB (0.3) (**e**), and SF–GG (0.3)–SB (0.4) (**f**).

Table 1 shows the remaining mass retention ratio of the hydrogels on various media. At the end of experiment (2 days), the SF–GG hydrogels had degradation of about 50% in PBS buffer, pH 7.4, but SF–GG (0.3)–SB hydrogels have a higher remaining mass retention rate value. Guar gum (GG) is a heteropolysaccharide which can react to water molecules in the buffer while sodium benzoate (SB), a structure composing an aromatic ring of benzoate, is a sodium salt of benzoic acid. Benzoic acid is generally not used directly due to its poor water solubility. This is because the hydrogel has a dense texture and protects the water absorbed into the hydrogel. Sodium benzoate usually acts as a food preservative and is also used as a preservative in medicines and cosmetics. In acidic conditions, the degradation value of the SF–based hydrogel was higher than in the PBS buffer. Moreover, the SF–based hydrogel is also dramatically degraded in NaOH solution with the lowest remaining mass. This indicated that NaOH could hydrolyze the peptide bonds in SF and the glycosidic bonds in GG as well as the H–bonds between SF and GG. In ethanol, the prepared hydrogels had degraded, but the remaining mass retention rate was slightly higher in value than in the PBS buffer. Ethanol, a polar solvent, can form hydrogen bonds with the hydrophilic groups of hydrogels. Therefore, it could penetrate into hydrogel networks and quickly degrade the hydrogel texture. However, the SF–based hydrogel displayed a slight swelling texture in NaCl solution. This might be affected by the porous network structure of SF, which can absorb the water. A more stable crystal structure was also formed inside the hydrogel because of some salt ions entering it. As a result, the hydrogel’s ability to absorb water is protected by its dense pores [18,36,37]. The hydrophilicity test indicated the water absorption of the hydrogel surfaces, which was expressed by the water contact angle (WCA). The WCA values increased when the GG content was increased (Table 2, Figure 4). The reason for this was that SF and GG linked together and decreased some gaps that water can penetrate. In addition, adding SB, a non-polar ring structure, resulted in increasing WCA values. From Table 2, the WCA values of SF–GG (0.3)–SB hydrogels increased gradually by increasing SB contents from 0.2 (63.75), 0.3 (70.28), and 0.4 (80.39), respectively. This increase in the WCA value indicates an increase in the hydrophobicity of the hydrogel which protected the water droplet that penetrated the inner layer with a small content [52,53].

### 3.3. Mechanical Properties of the Hydrogels

The mechanical properties of SF–based hydrogels are shown in Table 3. The highest tensile strength (53.3 MPa) and Young’s modulus (226.3 MPa) were found from the SF–GG (0.3)–SB (0.4) hydrogel, but elongation at break showed the lowest value (8.4%). Other hydrogels, SF–GG (0.3)–SB (0.3) and SF–GG (0.3)–SB (0.2), exhibited a gradually decreased tensile strength (48.1 and 46.4 MPa, respectively) and Young’s modulus (221.5 and 219.7 MPa, respectively). Without SB, the hydrogels showed both a lower tensile strength and Young’s modulus. However, elongation at break was higher for the SF–GG (0.3)–SB hydrogels. The SF–GG (0.3) hydrogel has the highest elongation at break of 27.0% while SF–GG (0.2) had the lowest value at 24.7%. In addition, the SF–GG (0.2) had the lowest tensile strength (32.9 MPa) and Young’s modulus (192.4 MPa). From the results, the tensile strength of the prepared hydrogels increased with an increase in both GG and SB contents. This indicated that the hydrogel–based SF formed with high GG and SB contents had higher mechanical properties. Adding SB reflected the rheological properties of the hydrogel since the SB structure is large from the benzene ring. Therefore, increased SB content resulted in the decreased elastic capacity of the hydrogels, which increased mechanical strength.

### 3.4. Clarification of Functional Groups

**Figure 5:**
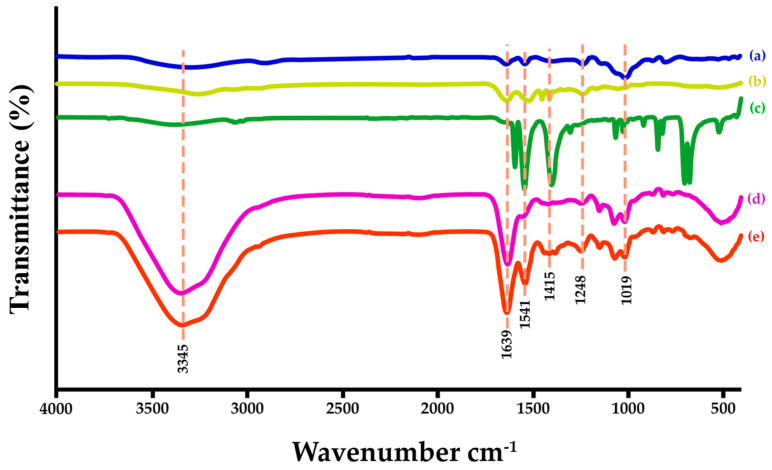
ATR–FTIR spectra of different materials; SF film (a), GG film (b), SB powder (c), SF−GG (0.3) hydrogel (d), and SF−GG (0.3)−SB (0.3)−hydrogel (e).

ATR–FTIR spectra of the SF–based hydrogels revealed their functional groups, shown in Figure 5. The significant functional groups of the SF (Figure 5 in (a)) are considered seriously at the absorption location, which is amide I (–CO stretching) at 1636 cm^−1^, amide II (–NH stretching) at 1517 cm^−1^, and amide III (–CN–stretching) at 1238 cm^−1^ [42]. The repeating units of polysaccharides contain many functional groups. Among their positive attributes are their biodegradability and biocompatibility. Drug delivery systems have made extensive use of GG, a naturally occurring heteropolysaccharide derived from seed gum [54]. Figure 5 in (b) is a FTIR spectrum of GG, which revealed the light absorption peaks of –CH stretching at 3000 and 2800 cm^−1^, ring stretching (1642 cm^−1^), symmetrical deformations of CH_2_ (1435 cm^−1^), highly coupled CC–O, C–OH, and C–O–C stretching modes (800–1200 cm^−1^), and galactose and mannose linkages (770–930 cm^−1^) [55,56,57]. The FTIR spectrum of the SF–GG (0.3) hydrogel is shown in Figure 5 in (d). The absorption peak at 3350 cm^−1^ for hydroxyl vibrations including intermolecular and intramolecular hydrogen bonding was broader and higher than that of SF. This indicated that characteristic absorption peaks of SF mixed with GG have some distinguishable changes due to interactions via their functional groups [15]. The other peaks for glucomannans polysaccharide (GG) were commonly found, such as 890 cm^−1^ for C–O–C stretching, 1019 and 1160 cm^−1^ for C–C and C–O–C asymmetric stretching, and 1415 cm^−1^ for CH_2_ bending [52]. The FTIR spectrum of SB powder is shown in Figure 5 in (c). The main absorption peaks appeared at 1404 cm^−1^ for aromatic C=C stretching, and 1546 and 1596 cm^−1^ for C=O stretching [58,59]. The spectrum of the SF–GG (0.3)–SB (0.3) hydrogel is shown in Figure 5 in (e). It has also been found that all the main functional groups of SF at amide I (1639 cm^−1^), GG at galactose and mannose linkages (700–1200 cm^−1^), and SB at aromatic structure (1404 cm^−1^), have dominantly appeared. The results indicate that all of them interacted, which enhanced the strength of the hydrogel.

### 3.5. Thermal Properties of Hydrogels

**Figure 6:**
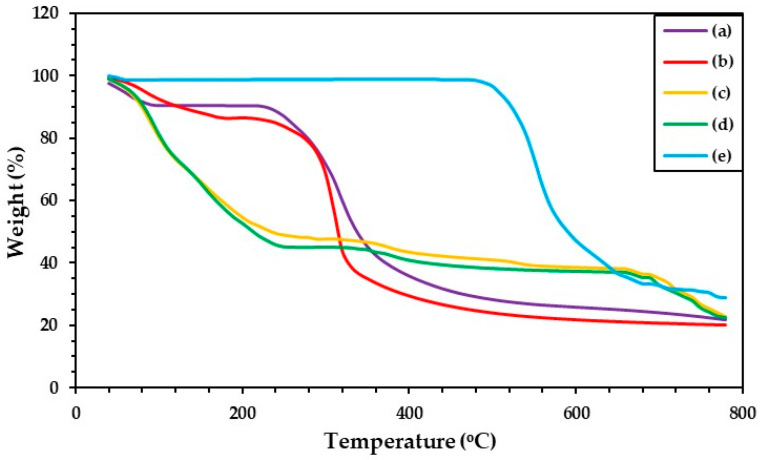
TG thermograms of different materials; SF film (a), GG film (b), SF–GG (0.3) hydrogel (c), SF–GG (0.3)–SB (0.3) hydrogel (d), and SB powder (e).

**Figure 7:**
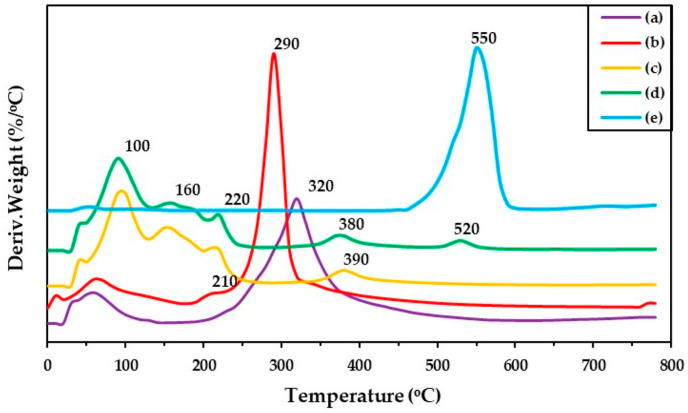
DTG curves of different materials; SF film (a), GG film (b), SF–GG (0.3) hydrogel (c), SF–GG (0.3)–SB (0.3)–hydrogel (d), and SB powder (e).

The thermal stability of all SF–based hydrogels was investigated by using a thermogravimetric analyzer (TGA). Figure 6 shows TG thermograms of the prepared hydrogels. The results indicated different peaks of weight loss, which was started by moisture loss at low temperatures (80–100 °C) [60]. SF shows a dominant peak of weight loss at 320 °C (Figure 6 in (a)). The decomposition peaks of the GG and mixed hydrogels appeared in at least two regions at 150–230 °C and 300–400 °C. The GG (Figure 6 in (b)) revealed two decomposition peaks at 210 and 290 °C, which were the decomposition peaks of the mannose and galactose parts in the GG structure. The SF–GG (0.3) (Figure 6 in (c)) and SF–GG (0.3)–SB (0.3) (Figure 6 in (d)) hydrogels showed three decomposition peaks according to the composition characteristics of the mixture materials. The SF–GG (0.3) hydrogel showed decomposition peaks at 152, 300, and 375 °C, whereas the SF–GG (0.3)–SB (0.3) hydrogel had decomposition peaks at 154, 375, and 531 °C. Among the hydrogels, weight loss was recorded at the initial stage for SF–GG (0.3), whereas no substantial loss was observed for the SB powder. The decomposition temperature of SB was observed after heating at 530 °C (Figure 6 in (d)). At 800 °C, the charred remains of all prepared hydrogels remained at about 20–30%; GG has remained charred residue of lower weight than others. Figure 7 illustrates the DTG curves of different materials, which exhibited the maximum decomposition temperature (T*_d_*_,*max*_). From the DTG results, SF showed the T*_d_*_,*max*_ at approximately 323 °C (Figure 7 in (a)), whereas SB’s (Figure 7 in (e)) was revealed at 553 °C. GG has a T*_d_*_,*max*_ at 311 °C with a shoulder peak at 227 °C (Figure 7 in (b)). The SF–GG (0.3) hydrogel (Figure 7 in (c)) had lower T*_d_*_,*max*_ values than the SF–GG (0.3)–SB (0.3) hydrogels (Figure 7 in (d)). Blending SB enhanced the T*_d_*_,*max*_ to 531 °C which is the dominant peak of SB. In addition, the T*_d_*_,*max*_ of the mixed hydrogels, especially at 375 °C, shifted from 311 °C for GG and 323 °C for SF. Some interactions between GG and SF would be formed and resulted in an increased T*_d_*_,*max*_. This interaction helped to increase the thermal stability of the hydrogels [61,62]. Conversely, no interaction between SF and SB was observed since the T*_d_*_,*max*_ of the mixed hydrogel was lower in value compared with SB powder. In addition, the T*_d_*_,*max*_ at 210–220 °C was the decomposition temperature of glycerol [43], which was used as a plasticizer. Table 4 summarizes the effects of some parameters on the thermal behavior of all materials and hydrogels.

## 4. Conclusions

In conclusion, SF–based hydrogels were successfully prepared by simple evaporation technique. The outcomes of the hydrogels demonstrated suitable characteristics for further applications such as ease of use, mechanical and thermal properties, and economy of combining materials. The constructed SF–based hydrogels have moderate transparency and light transmittance, with some different appearances among mixed GG and SB. SF–GG (0.3) showed the best ratio which has smoother surfaces and is more rigid. However, the dense texture of the hydrogel had resulted from mixing SB. SF–GG (0.3)–SB (0.4) hydrogels had the highest tensile strength, thermal stability, and WCA value, but a decreased elongation at break. ATR–FTIR results revealed that the intermolecular and intramolecular bonding was broader and higher in SF after mixing GG and SB. The interaction also resulted in an increase in the T*_d_*_,*max*_ of the hydrogels. This suggested that the structure and functional groups of the substances impacted on the thermal and mechanical properties of the hydrogels. SF–GG–SB hydrogels showed high stability on various solvents, resulting in structural stability for application while they could be environmentally degraded. Even though further studies are still needed to confirm its practical use, our study shows that SF–based hydrogel might be used in specific applications including wound healing and tissue engineering.

## Figures and Tables

**Table 1:** Light transmittance and degradation of hydrogels in different media.

Samples	T660 (%)	Remaining Mass Retention Rate (%) (at 2 Days)				
		PBS pH 7.4	1.0 M HCl	1.0 M NaOH	95% Ethanol	1.0 M NaCl
SF–GG (0.2)	65.30 ± 1.42	50	0	0	55	105
SF–GG (0.3)	60.22 ± 2.66	55	3	3	65	105
SF–GG (0.4)	51.80 ± 1.71	65	5	5	75	105
SF–GG (0.3)–SB (0.2)	24.80 ± 2.05	70	10	12	80	102
SF–GG (0.3)–SB (0.3)	10.60 ± 1.48	75	15	13	85	102
SF–GG (0.3)–SB (0.4)	4.50 ± 2.64	80	20	15	90	102

**Table 2:** Water contact angle (WCA) of the prepared SF–based hydrogels.

Samples	WCA (°)	Figure 4
SF–GG (0.2)	21.50	a
SF–GG (0.3)	42.35	b
SF–GG (0.4)	54.05	c
SF–GG (0.3)–SB (0.2)	63.75	d
SF–GG (0.3)–SB (0.3)	70.28	e
SF–GG (0.3)–SB (0.4)	80.39	f

**Table 3:** Mechanical properties of the prepared SF–based hydrogels.

Samples	Force at Peak (N)	Tensile Stress (MPa)	Elongation at Break (%)	Young’s Modulus (MPa)
SF–GG (0.2)	150.6	32.9	24.7	192.4
SF–GG (0.3)	174.7	38.0	27.0	202.2
SF–GG (0.4)	186.1	40.8	18.5	215.8
SF–GG (0.3)–SB (0.2)	205.7	46.4	12.3	219.7
SF–GG (0.3)–SB (0.3)	207.4	48.1	10.8	221.5
SF–GG (0.3)–SB (0.4)	215.5	53.3	8.4	226.3

**Table 4:** Thermal behaviors of SF–based hydrogels.

Hydrogels	Onset of Decomposition (°C)	T*_d_*_,_*_max_* (°C)	Charred Residue Weight at 800 °C (%)
SF film	95	323	22
GG film	90	227, 311	20
SB powder	-	553	22
SF–GG (0.3)	90	152, 300, 375	22
SF–GG (0.3)–SB (0.3)	90	154, 375, 531	22

## Data Availability

Data is contained within the article.
